# Previous Use of Combined Oral Contraception in High Complexity Assisted Reproduction Treatments in Protocol with Oral Progestin - Previous use of COC and ART

**DOI:** 10.5935/1518-0557.20240058

**Published:** 2024

**Authors:** Daniel Henrique Braga Vidal, Fabiana Lanoli Gentil, Erik Montagna, Caio Parente Barbosa, Renato de Oliveira

**Affiliations:** 1 Centro Universitário FMABC, Departamento de Saúde Coletiva - Setor de Reprodução Humana - Instituto Ideia Fértil - Santo André, São Paulo, Brazil; 2 Centro Universitário FMABC, Pós-graduação - Santo André, São Paulo, Brazil

**Keywords:** combined oral contraceptives, *in vitro* fertilization, infertility, progestin, ovulation induction

## Abstract

**Objective:**

To evaluate the impact of prior use of combined oral contraceptives in assisted reproduction techniques with ovulation blockade by oral progestin.

**Methods:**

Retrospective cohort study with a single-center convenience sample of patients treated between 2018 and 2021. Two groups were compared: with and without a history of combined oral contraceptives (comparator). The clinical variables were age, body mass index, type of infertility and smoking. Regarding treatment, antral follicle count; follicles >14 mm; oocytes in metaphase I and II; number of embryos; days of treatment; total dose of medication, chemical and clinical pregnancy rate and delivery after 1st embryo transfer. Absolute and relative frequencies were used for the qualitative variables; means, medians and t-test for the quantitative ones. Association between qualitative variables used the Chi-square test and, for quantitative variables, the Mann-Whitney test (p<0.05). The statistical program used was Stata 16.0.

**Results:**

Among 407 medical records, 351 were included (combined oral contraceptive=243 and comparator=108). The combined oral contraceptive and the comparator groups had, respectively, mean (SD±) age 38.2 (4.5) and 38.2 (4.5) years; chemical pregnancy rates of 30.5% and 29.6% (p=0.281); clinical pregnancy rates of 24.8% and 24.1% (p=0.313) and abortion, 5% and 4.6% (p=0.544). The median time on combined oral contraceptives was 6 years.

**Conclusions:**

Previous use of combined oral contraceptives did not impact reproductive results in relation to the comparator group in patients undergoing assisted reproduction techniques in protocols with oral progestin.

## INTRODUCTION

Currently, the time between the first sexual intercourse and gestational desire has increased, a fact that causes the use of a contraceptive method (CM) to be prolonged. Furthermore, the secondary benefits of hormonal methods also motivate their indication (Borges & Schor, 2005; Bahamondes & Bahamondes, 2014). The combined oral contraceptive (COC) method stands out, which depends on the eligibility criteria of the World Health Organization (WHO) (World Health Organization, 2018).

Regarding the impact on fertility, Chasan-Taber *et al.* (1997) suggested an adverse effect of previous long-term use of COC. Concerning ovarian reserve, there was a reduction, possibly functional, in antral follicle count (AFC) and in the value of Anti- Müllerian Hormone (AMH), which interruption for 3 to 6 months could cause a reversal of this situation (Petersen *et al.*, 2015).

Possible endometrial effects of progestin and lower levels of luteinizing hormone (LH) at the beginning of the controlled ovarian stimulation (COS) cycle would justify this deleterious effect (Ciari Jr. *et al.*, 1972; Griesinger *et al.*, 2008; 2010). However, this is not a consensus, as reported in other studies, in which previous use of COC did not affect pregnancy and live birth rates (Garcia-Velasco *et al.*, 2011; Hauzman *et al.*, 2013; Garcia-Velasco & Fatemi, 2015; Griesinger *et al.*, 2015; Farquhar *et al.*, 2017).

In 2023, Homminga *et al.* (2023) reported in a retrospective study with patients undergoing preimplantation genetic testing for monosomy (PGT-M), a significant association between lower endometrial thickness and previous use of COC. Additionally, it is unknown whether prolonged use of COC could have any epigenetic impact on reproductive outcomes, interfering, for example, with oocyte quality (Dupont *et al.*, 2009; Parihar *et al.*, 2010).

In 2015, a new AFC protocol emerged demonstrating the possibility of blocking ovulation with oral progestin (OP) (Kuang *et al.*, 2015). Despite the ease of dosing and the lower cost compared to medications used in traditional protocols (Oliveira *et al.*, 2021), the legitimacy of using new strategies requires an understanding of their real cost-benefit. In this context, the controversial topic about the impact of previous use of COC and its relationship with new highly complex assisted reproductive treatment (ART) strategies contribute to improving reproductive counseling, a fundamental strategy to minimize the emotional exhaustion inherent to infertility treatments (Ciari Jr. *et al.*, 1972).

The objective of this study was to evaluate the impact of previous use of COC on the reproductive results of patients undergoing ART with COS using OP. The null hypothesis is that previous use of COC does not impact ART results and reproductive outcomes. The alternative hypothesis is that previous COC use worsens both ART results and reproductive outcomes.

## MATERIAL AND METHODS

The STROBE recommendations were followed in this study (Malta *et al.*, 2010), which was approved by the Research Ethics Committee of the Centro Universitário FMABC (CAAE: 90584718.8.0000.0082 and opinion number 3.076.655).

This is a retrospective cohort study with a convenience sample from a single center, Instituto Ideia Fértil, of patients undergoing high-complexity ART between 2018 and 2021, the period of institutional introduction of the COS protocol with OP to block ovulation. Two groups were considered: with previous use of COC and without previous use of COC (comparator group).

The inclusion criteria were the presence of information on age, body mass index (BMI), female smoking (pack-years), cause of infertility (anovulation, endometriosis, peritoneal tube factor, uterine factor - myomatosis, immunological - chronic diseases, maternal age (>35 years), genetic causes (chromosomal alterations or gene diseases described by a geneticist), male factor (concentration/ml less than the 5th percentile and asthenoteratozoospermia) (World Health Organization, 2021) and idiopathic.

The description of the characteristics of the patients’ treatments considered information regarding AFC, follicles larger than 14 mm formed at the end of COS (preovulatory follicles), FORT (Follicular Output RaTe) index, whose calculation is characterized by the number of pre-ovulatory follicles x 100/AFC (Carosso *et al.*, 2022) number of oocytes recovered, number of oocytes in metaphase I (MI) and II (MII), number of fertilized oocytes, number of pre-embryos (D3), formed blastocysts and top qualities blastocysts, number of days of treatment, total dose of medication and pregnancy status.

Patients undergoing ART were considered with previous exclusive use of COC on a continuous basis or absence of use of any previous hormonal contraceptive methods; submission to protocols using dosages of 100, 150 or 200 IU of medications with recombinant FSH - FSHr (Menopur^©^, Rekovelle^©^, Ferring BV; Gonal^©^, Merck Sharp & Dohme BV, Holanda; Fostimon^©^, IBAS, Itália, Puregon^©^, Organon, Holanda), associated with OP (didrogesterona; Duphaston^©^ Abbott); COC interruption time compatible with infertility time.

Patients underwent IVF (in vitro fertilization) / ICSI (intracytoplasmic sperm injection) according to clinical indications and failure of low-complexity treatment, defined as absence of pregnancy after three attempts (Practice Committee of the American Society for Reproductive Medicine, 2013). Exclusion criteria were incomplete medical records, use of other contraceptive methods, patients undergoing EOC for fertility preservation, history of radiotherapy and chemotherapy and canceled cycles (Barbosa *et al.*, 2014).

The primary variable was the previous use of COC. The secondary variables were the average female age (years), duration of contraception use (years), duration of infertility (years), number of medication days, total dose of medication (IU), antral follicle count, number of dominant follicles, mean FORT value, mean MI, mean MII and total number of embryos formed.

The primary outcomes were chemical pregnancy rate (*pregnancy Beta HCG - preg_beta*), clinical pregnancy rate (*pregnancy_clinic* - *preg_clin*) and abortion rate (*abortion - abort*).

In relation to the group with previous use of COC, the time from interruption until the attempt at pregnancy was considered as the time of infertility.

COS used 100UI of FSHr daily, started on the second or third day of the menstrual cycle, only if age ≤35 years and without previous ovarian inductions or surgeries and total AFC greater than 15; and 150 or 200IU of FSHr per day, for patients with low response to previous COS, age >35 years, single ovary or previous ovarian surgery, using conventional two-dimensional transvaginal ultrasound at 7Mhz (Philips^®^). AFC was performed on each ovary and antral follicles were considered if larger than 2 mm and smaller than 10mm (Broekmans *et al.*, 2010).

Early blocking of ovulation occurred with 2 tablets of dydrogesterone (Duphaston^®^ 10mg; Abott) once a day, started orally from the day the use of exogenous gonadotropins began until ovulation was triggered with 2 ml of ag-GnRH (Triptorelin acetate, Gonapeptyl Daily^®^ 0.1 mg; Ferring) when a minimum of 3 follicles reached between 17 and 22 mm. After 35 hours, ovarian puncture took place.

Chemical pregnancy was considered when there was a positive value and confirmed by serum measurement of the beta fraction of hCG (βhCG) between the 10th and 12th day after the first embryo transfer. If a gestational sac was visualized on transvaginal ultrasound, it was considered a clinical pregnancy (ASRM - American Society for Reproductive Medicine, 1996). Abortion, in this study, was considered the arrest of embryonic evolution until the first trimester of pregnancy.

The classification of ovarian response as ovarian hyperstimulation syndrome (OHSS) followed the criteria of Golan *et al.* (1989).

According to the degree of nuclear maturation, oocytes were classified as metaphase II (MII) or non-metaphase II (Alpha Scientists in Reproductive Medicine and ESHRE Special Interest Group of Embryology, 2011).

The classification of blastocysts was in accordance with that proposed by Gardner *et al.* (2000), based on the morphological aspect of their expansion, which varies from 1 to 6, on the characteristics of the internal mass and trophectoderm, with classification A, B or C. Those with a rating ≥3BB (top quality) are considered to be of good quality.

Qualitative variables are presented as absolute and relative frequencies and quantitative variables as means and medians, 25th and 75% percentiles and confidence intervals (CI) and t-test. To analyze the association between qualitative variables, the Chisquare test was used, and for quantitative variables, due to the non-normal distribution of the data (Shapiro-Wilk, *p*<0.05), the Mann-Whitney test was used. The confidence level was 95%. The statistical program used was Stata 16.0.

## RESULTS

**Figure 1:**
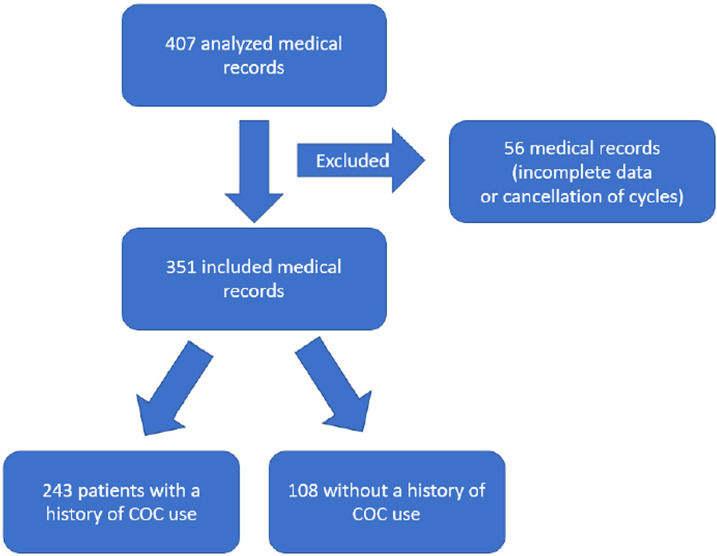
Flowchart of selection of patients undergoing ART with OP in relation to previous use of COC. Source: author. Legend: COC (combined oral contraceptive); ART: assisted human reproduction techniques.

Patient selection occurred by evaluating electronic medical records as described in the flowchart in Figure 1.

The characterization data of the group of patients undergoing ART with OP in relation to previous use of COC are described in Table 1. The median time using COC is 6 years. Among the patients with genetic alterations, all had chromosomal abnormalities identified by the G-band karyotype of peripheral blood, routinely requested in this service. Regarding immunological diseases, 12 had anti-nuclear factor (ANF) (>1:320), 2 with Systemic Lupus Erythematosus and 1 with Rheumatoid Arthritis.

**Table 1:** Characterization of patients undergoing ART with OP in relation to previous use of COC.

Variables	Groups (n)	
	COC n=243	Comparator n=108
Maternal age (years) (µ±sd)	38.18±4.49	37.98±4.57
Maternal BMI (kg/m^2^) (µ±sd)	25.67±3.86	25.99±3.25
COC time (years) (µ±sd)	7.11±5.72	-
Infertility time (years) (µ±sd)	3.81±2.84	2.89±3.4
Paternal age (years) (µ±sd)	40.42±5.95	40.72±7.36
Paternal BMI (kg/m^2^) (µ±sd)	28.17±4.39	28.31±3.50
Maternal smoking (years/pack)	12	7
Infertility factors n (%) Anovulation Endometriosis Peritoneal tube Uterine Immunological Maternal age (>35 years) Genetic Male idiopathic	26 (10.70) 29 (11.93) 40 (16.46) 38 (15.64) 12 (4.94) 161 (66.26) 13 (5.35) 113 (46.50) 17 (7.05)	14 (12.96) 6 (5.56) 13 (12.04) 13 (12.04) 3 (2.78) 65 (60.19) 5 (4.63) 53 (49.07) 9 (8.41)

COC (combined oral contraceptive); BMI (body mass index).

The characterization of the variables related to COS and laboratory results of the comparator groups and those with previous use of COC is shown in Table 2. The average number of D3 pre-embryos formed in the comparator group was 2.25 (SD±2.93) and in the COC group it was 2.9 (SD±3.27) with *p*=0.035. The other variables did not show a statistically significant variation between both groups.

**Table 2:** Characterization of COS response variables and laboratory results of patients undergoing ART with OP in relation to previous COC use.

Variables	Groups		
	COC	Comparator	*p*
	n=243	n=108	
Medication days (µ±sd)	10.40±2.50	10.49±8.36	0.083
Total dose of medication (IU) (µ±sd)	1914.47±565.15	1955.33±1280.44	0.525
Antral follicle count (µ±sd)	10.13±9.42	9.84±5.93	0.859
Pre-ovulatory follicles (µ±sd)	7.1±4.97	6.72±4.85	0.464
FORT (%) index (µ±sd)	69.43±28.68	64.68±28.51	0.099
MI (µ±sd)	0.38±0.87	0.55±1.47	0.420
MII (µ±sd)	5.6±4.39	5.88±4.65	0.577
Oocytes captured (µ±sd)	6.16±4.78	5.81±4.19	0.633
Pre-formed embryos (µ±sd)	3.44±3.12	3.22±2.92	0.569
Pre-embryos at D3 (µ±sd)	2.9±3.27	2.25±2.93	0.035
Blastocysts (µ±sd)	1.69±2.30	2.22±2.75	0.050
Blastocysts top quality (µ±sd)	0.92±1.70	1.15±1.84	0.280

COC (combined oral contraceptive); UI (International Units); FORT (follicular output rate); MI (metaphase I); MII (metaphase II); D3 (day 3 of embryonic cleavage).

Table 3 presents the primary outcomes rate of chemical pregnancy, clinical pregnancy and abortion, in addition to the OHSS complication between the comparator groups and with previous use of COC. There was no statistically significant difference between both groups.

**Table 3:** Characterization of the clinical outcomes of patients undergoing ART with OP in relation to previous use of COC.

Variables	Groups		
	AOC	Comparator	p
	n=243	n=108	
Beta-HCG pregnancy n (%) No Yes	169 (69.55) 74 (30.45)	76 (70.37) 32 (29.63)	0.281
Clinical pregnancy n (%) No Yes	179 (75.21) 64 (24.79)	82 (75.93) 26 (24.07)	0.313
Abortion n (%) No Yes	227 (94.98) 16 (5.02)	103 (95.37) 5 (4.63)	0.544
OHSS n (%) No Yes	219 (91.63) 24 (8.37)	98 (90.74) 10 (9.26)	0.750

COC (combined oral contraceptive); Beta-HCG pregnancy (chemical pregnancy); OHSS: ovarian hyperstimulation syndrome.

**Figure 2:**
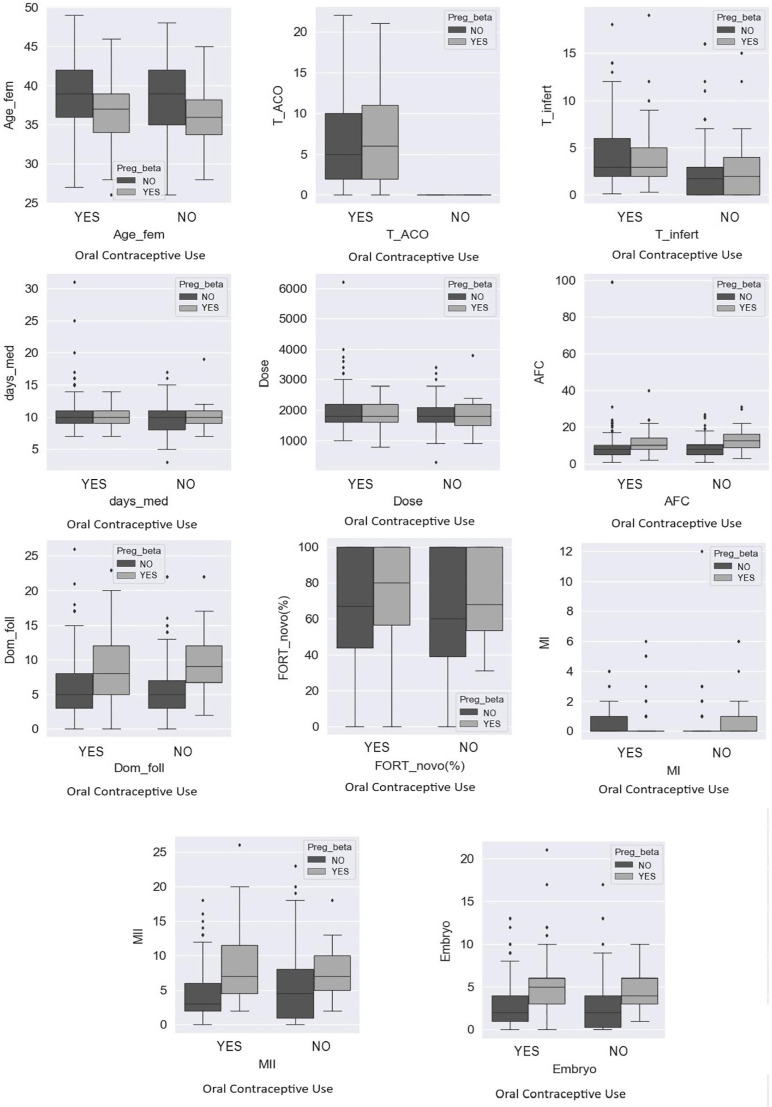
Comparative analysis between the primary and secondary outcomes in relation to the biochemical pregnancy rate between the comparator and with COC use groups.

**Figure 3:**
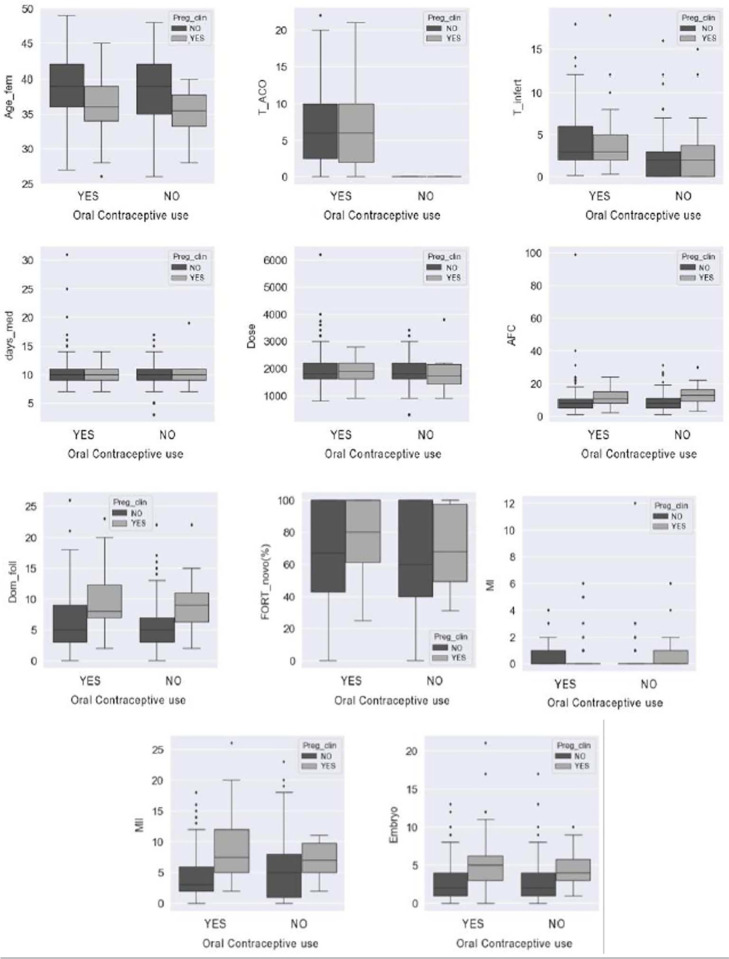
Comparative analysis between the primary and secondary outcomes in relation to the clinical pregnancy rate between the comparator and non-COC use groups. Legend: Preg_clin (clinical pregnancy); Age fem (feminine age); TAOC (COC use time); T_infert (infertility time); days_med (medication days); AFC (antral follicle count); Dom_fol (pre-ovulatory follicles); FORT (*follicular output rate*); MI (metaphase I); MII (metaphase II); Embryo (number of total embryos); Previous use of COC (YES or NO).

**Figure 4:**
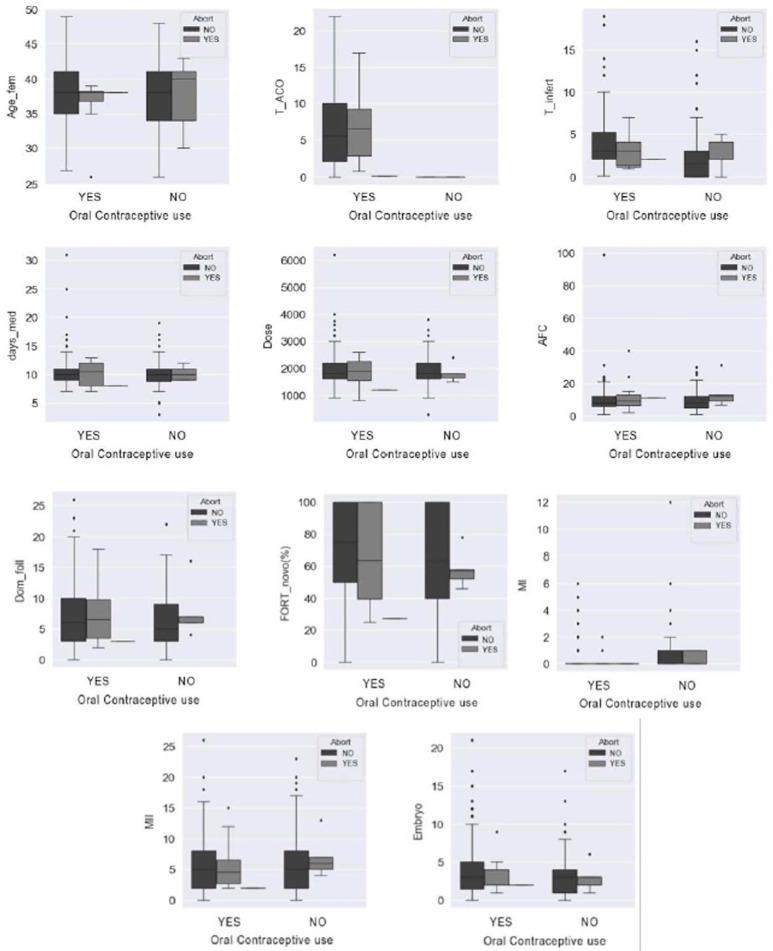
Comparative analysis between the primary and secondary outcomes in relation to the abortion rate between the comparator groups and those without COC use. Legend: Abort (abortion); Age fem (feminine age); TAOC (COC use time); T_infert (infertility time); days_med (dias de medicação); AFC (antral follicle count); Dom_fol (preovulatory follicles); FORT (follicular output rate); MI (metaphase I); MII (metaphase II); Embryo (number of total embryos); Previous use of COC (YES or NO).

The primary clinical outcomes chemical pregnancy (*preg_beta*), clinical pregnancy (preg_clin) and abortion were compared, respectively, in Figures 2, 3 and 4 with the means of the variables female age (*Age_fem*), time of contraceptive use (T_COC), time of infertility (*T_infert*), number of days of medication (*days_med*), total dose of medication (dose), antral follicle count (AFC), number of dominant follicles (Dom_fol), mean FORT value (*FORT_mean*), MI, MII and total number of embryos formed (Embryo) both in the group with previous use of COC (YES) and in the comparator group (NO). In all associations demonstrated in relation to the three outcomes there was no statistically significant difference.

The comparative analysis between the clinical pregnancy rate of the comparator and COC groups in relation to the different age groups is described in Table 4, without showing a statistically significant difference between both groups.

**Table 4:** Clinical pregnancy rates of the group with and without COC in relation to the age groups <35 years, between 35 and 40 years and >40 years.

Age group	Groups		
	COC	Comparator	p
< 35 years n (%)	18 (28.12)	11 (42.31)	0.278
35-40 years n (%)	34 (53.13)	13 (50)	
> 40 years n (%)	12 (18.75)	2 (7.69)	

COC (combined oral contraceptive).

## DISCUSSION

The results presented in this study show that there was no statistically significant difference between the comparator group and the one with previous use of COC in relation to the rate of chemical and clinical pregnancy and abortion. This provides valuable and reassuring information to guide patients regarding this frequent question, considering the wide use of COCs in the population.

In line with these findings, in 2023, considering a group of women with infertility, Siegel *et al.* (2023) evaluated ovarian reserve markers such as AMH, FSH and AFC in long-term (≥2 years), short-term (<2 years) or no history of hormonal contraceptive users, reporting that, although long-term users used more ART, in particular IVF, overall conception rates and live birth outcomes among ART users were not significantly affected by prior COC use.

The analysis of the other variables in relation to the primary outcomes did not show a statistically significant difference, except for the number of pre-embryos at D3 for the COC group of 2.90 (±3.27) vis-a-vis the comparator group of 2.25 (±2.93); with *p*=0.035. However, the average number of cryopreserved blastocysts was 1.69 (±2.30) in the COC and 2.22 (±2.75) in the comparator, with *p*=0.05. This reversal in embryonic evolution could be a reflection of a convenience sample, a possible selection bias. However, the relationship between the total number of embryos formed and the chemical and clinical pregnancy rates were equivalent, suggesting that the previous use of COC does not present a statistically significant difference to highly complex ART.

The total doses of gonadotropins in the COC group was 1914.47IU (±565.15), and in the comparator group it was 1955.33IU (±1280.44); likewise the average number of days of medication was respectively 10.40 (2.50) and 10.49 (8.36) days. This is compatible with other studies (Massin, 2017; Caetano *et al.*, 2022), differing only from Oliveira *et al.* (2021), who reported an average of 12.8 days in an COC with OP block; but this is a pilot study for oncological preservation of fertility.

On the other hand, the average number of oocytes captured and the number of embryos formed are compatible with values from other studies (La Marca & Capuzzo, 2019; Huang *et al.*, 2021; Caetano *et al.*, 2022).

Recently, Avramenko & Flanagan (2023) described the possibilities of epigenetic influence with the previous use of COC, which could play a fundamental role in long-term chemoprevention of the development of ovarian cancer. However, despite the objective of the present study not having an epigenetic focus, the analogy with a possible effect on fertility parameters with previous use of COC did not demonstrate an improvement in AFC. Likewise, the response to treatment did not improve both in terms of COS, comparing the total dose of gonadotropins and the number of days of medication; and in the response to the number of dominant follicles, FORT index, number of mature oocytes and number of embryos formed. Therefore, there is no evident epigenetic impact on the reproductive response in this study group. However, it is noteworthy that studies with specific designs for this issue are necessary.

Regarding the time of COC use, a UK cohort study, including 339,000 womenyears of observation for those who have never used COC and 744,000 women-years for those who have already used COC, aiming to identify cancer risk, did not find an association to an overall increased risk of this disease; in fact, it could even produce a protective effect. The average time of use was 44 months, almost 4 years (Hannaford *et al.*, 2007). By analogy, although there is no study that evaluates the protective effect with a significant number of patients undergoing highly complex ART with OP, the median time in the present study was 6 years, a period supposedly adequate to impact the body and, consequently, affect possible reproductive outcomes. During the use of COC there is an approximate 20% reduction in the ovarian reserve values in AFC and in the HAM value, but this is reversed after its interruption (Ciari Jr. *et al.*, 1972). For women without a history of infertility and planning a pregnancy, Linn *et al.* (1982) demonstrated that there is no significant association between COC use interruption and conception. On the other hand, studies have described that hypothalamic suppression caused by COC could compromise follicular maturation up to 3 months after stopping contraception (Pardthaisong & Gray, 1981; Harlap & Baras, 1984; Bracken *et al.*, 1990). A prospective observational study of 2,874 women also did not identify a difference in long-term pregnancy rates after discontinuing COCs (Berglund Scherwitzl *et al.*, 2019). The data from the present study, considering an average infertility time of 3.81 (±2.84) years in the COC group, suggest that this time was sufficient to minimize a possible deleterious effect. This is not a cut-off score, something limited by the nature of the study. The aim is to demonstrate that there was no significant association between the time of COC use cessation (time of infertility) and laboratory and therapeutic reproductive outcomes in a group of patients undergoing ART.

Regarding the causes of infertility, Chasan-Taber *et al.* (1997) even without showing statistical relevance, reported an increased risk of ovulatory disorders in patients with previous use of COC. Fraser & Weisberg (1982) on the other hand, suggested that the use of COC would be a treatment for ovulatory causes. In the present study, there was a higher number of patients with ovulatory causes in the group with a history of COC use compared to the comparator group (Table 1). However, the restricted number of patients, 26 and 14 respectively, limits the evaluation.

Since the 90s, research has focused mainly on the relationship between the use of COC and TRA through pre-treatment (prime); that is, the use immediately before COS, mainly aiming at follicular synchronization (Griesinger *et al.*, 2008; 2010; 2015; Kim *et al.*, 2009; Garcia-Velasco *et al.*, 2011; Hauzman *et al.*, 2013; Garcia-Velasco & Fatemi, 2015; Farquhar *et al.*, 2017; Xu *et al.*, 2019; Lu *et al.*, 2020; Montoya-Botero *et al.*, 2020). Thus, there is a lack of literature regarding the prior use of COC in patients before the diagnosis of infertility.

According to data from Red Latinoamericana de Reproducción Asistida (RedLara), published in 2020; 87,732 ART cycles were registered, 46% of which came from Brazil. In general, there was an increase of around 34% in ART cycles for patients aged 40 years or older, and a drop of 24.7% if aged 34 years or less. Endometriosis was diagnosed in 28.3% of the cases. Frozen-thawed embryo transfer (TED) represented 66.6% of all transfers, with a pregnancy/transfer rate of 29.0%, significantly higher than 23.9% after fresh embryo transfers at all ages (*p*<0.0001). Pregnancy/elective single embryo transfer rates by age ranged from 33.5 to 41.7% in patients aged ≤35 years, 26.3 to 35.4% in those aged 35 to 40 years, and 13.5 to 26% for ages over 40 years of age (Zegers-Hochschild *et al.*, 2023). Data compatible with those of the present study. Although the pregnancy rate in the COC group was lower than expected for the group under 35 years of age, the limitation of a convenience sample and the care in analyzing subgroup results in this situation are highlighted. Furthermore, it is noteworthy that the treatment took place during a Covid-19 pandemic phase, when reproductive results could be affected. The fact of only including the first TED, instead of considering the pregnancy rate per cycle, could also impact the results presented.

The fear about the impact of previous use of COC on fertility was evidenced in a systematic review, published in 2021, which, based on a selection of 42 articles, considered this as one of the eight main categories that justify the reasons for rejecting hormonal contraception in western women (Oliveira *et al.*, 2021). This legitimizes further research that can answer this question, especially for patients undergoing ART.

With the emergence in 2015 of protocols using OP to block the LH surge, considering the ease of dosing and the lower cost compared to traditional blocks with gonadotropinreleasing hormone analogues, there is a tendency to expand this use in ART (Chen *et al.*, 2024). The present study is the first, to our knowledge, to evaluate the effect of previous COC use on highly complex ART outcomes involving only COS protocols with OP block.

Limiting factors of the study include its retrospective nature, the scarcity of considerations such as ethnicities, the correct use of COC, the lack of differentiation between the possible combinations of COC, impacts of male factor on reproductive outcomes and the lack of a sample power calculation, since it is a convenience sample. However, in relation to male factors, considering that they are associated with 40 to 50% of all causes of infertility, a similar distribution is believed in both groups evaluated (Carson & Kallen, 2021).

The external validity of the data is restricted to a group of patients undergoing ART with COS with OP block, hence the need for further studies on the topic, including prospective and randomized studies. However, the lack of a statistically significant difference between both groups, especially in relation to the main outcomes, contributes to reproductive counseling and the maintenance of the search for friendlier protocols, aimed at more humanized treatment.

The pioneering nature of this study, to our knowledge, opens new perspectives on a real-life issue for women, often discussed in doctors’ offices, characterized by the fear that previous use of COC has impacted reproductive capacity, something not found in the results of this study. This could reduce the anxiety of women involved in the highly complex ART process, in addition to optimizing guidance for COC users planning a future pregnancy.

## CONCLUSION

The history of COC use by patients undergoing ART with COS using OP to block ovulation does not impact reproductive outcomes in relation to treatment results, as well as gestational outcomes, rates of chemical and clinical pregnancy and miscarriage.
